# Attenuation of Multiple *Vibrio parahaemolyticus* Virulence Factors by Citral

**DOI:** 10.3389/fmicb.2019.00894

**Published:** 2019-04-25

**Authors:** Yi Sun, Du Guo, Zi Hua, Huihui Sun, Zhanwen Zheng, Xiaodong Xia, Chao Shi

**Affiliations:** ^1^ College of Food Science and Engineering, Northwest A&F University, Yangling, China; ^2^ Sino-US Joint Research Center for Food Safety, Northwest A&F University, Yangling, China

**Keywords:** citral, *Vibrio parahaemolyticus*, quorum sensing, biofilm, anti-virulence

## Abstract

Citral was known as a widely used food additive with antimicrobial activity; however, whether it can be a potential therapy for controlling bacterial virulence with less risk of antimicrobial resistance remains to be investigated. Herein, we demonstrated that *Vibrio parahaemolyticus* virulence factors that contribute to infection were effectively inhibited to different degrees by sub-inhibitory concentrations (3.125, 6.25, and 12.5 μg/ml) of citral. Citral exerted strong inhibition of autoinducer-2 production and adhesion to Caco-2 cells. Biofilm formation of *V. parahaemolyticus* was effectively decreased by citral at 30°C and 20°C. Moreover, citral repressed the transcription of genes related to flagella biosynthesis, biofilm formation, type III secretion effectors, and antibiotic resistance, as well as genes contributing to the regulation of quorum sensing and toxin production. Therefore, citral could effectively attenuate multiple virulence properties of *V. parahaemolyticus*, and its effect on *in vivo* infection by *V. parahaemolyticus* needs further investigation.

## Introduction

The increasing occurrence of disease outbreaks caused by antibiotic-resistant pathogens is becoming a major cause of mortality worldwide (Letchumanan et al., 2015). Therefore, there is an urgent need to identify novel and alternative strategies to control disease outbreaks. Anti-virulence therapies aim to inhibit the specific functions of pathogens that are required to cause infection (Rasko and Sperandio, 2010). As virulence factors are not necessary for bacterial survival, anti-virulence therapies tend to be less prone to the development of resistance and have less impact on neutral and beneficial host bacteria compared with traditional antimicrobials (Rasko and Sperandio, 2010).

*Vibrio parahaemolyticus* is a halophilic, motile, curved gram-negative bacterium that is frequently associated with foodborne outbreaks of disease, usually following the consumption of contaminated seafood such as raw or improperly cooked shellfish and ready-to-eat foods (Wu et al., 2014; Xie et al., 2016). *V. parahaemolyticus* contains various virulence factors responsible for several distinct diseases, including wound infections, human acute gastroenteritis, septicemia, and even death and is therefore a significant cause for concern regarding seafood safety (Yeung et al., 2002).

The virulence factors of *V. parahaemolyticus* are complex and interactive. The bacterium readily forms biofilms on food-processing surfaces such as kitchen cutting boards and stainless steel and can adhere to human intestinal cell lines, contributing to cross-contamination and the ensuing diseases (Chiu et al., 2006; Mizan et al., 2016). During the establishment of infection, motility (swimming and swarming) is an important function of biofilm formation and adhesion (Zhu et al., 2013). Following intimate contact with the host cells, *V. parahaemolyticus* then secretes toxin proteins and delivers type III secretion system (T3SS) effectors into the host cell cytoplasm, inducing cytotoxicity and bacterial enterotoxicity (Makino et al., 2003; Park et al., 2004). In *V. parahaemolyticus*, quorum sensing (QS) is a cell-to-cell communication system that responds to fluctuations in cell population density through the secretion of autoinducer-2 (AI-2) (Mizan et al., 2016). Once AI-2 has reached a critical threshold concentration, the QS system begins to induce the expression of several virulence factors, including motility (Zhu et al., 2013), biofilm formation (Mizan et al., 2016), adhesion (LaSarre and Federle, 2013), T3SSs (Henke and Bassler, 2004), and toxin production (Zhu et al., 2002).

Essential oils have long been recognized as eco-friendly anti-microbial materials with low mammalian toxicities (Isman, 2000). Citral (3,7-dimethyl-2,6-octadienal) is the principal component of lemongrass oil and has strong antimicrobial activity (Adukwu et al., 2016). Additionally, citral is widely used as a health-promoting food additive for human and animal (FDA, GRAS, 21 CFR 182·60). Because of its strong antimicrobial activity and wide application in food products, the potential of citral to affect bacterial virulence factors should be recognized.

To date, anti-QS approach is one of the most intensively studied in anti-virulence therapies (LaSarre and Federle, 2013). In addition, therapies aimed at attenuating pili, secretion systems, or toxin production have also been reported, respectively (Rasko and Sperandio, 2010). However, the mechanism of *V. parahaemolyticus* pathogenesis is complicated and remains unclear; thus, alternative approaches that interfere with multiple virulence factors should be given more attention. The aim of the current study was to investigate the effect of citral at sub-inhibitory concentrations on the various virulence properties of *V. parahaemolyticus*. The effects of citral on QS, motility, biofilm formation, and adhesion of *V. parahaemolyticus* were investigated, and the effects of citral on the transcription of virulence-associated genes and antibiotic resistance genes were also examined.

## Materials and Methods

### Reagents

Citral (CAS:5392-40-5) was obtained from the Chengdu Must Bio-technology Co., Ltd. (Chengdu, Sichuan, China) at a high-performance liquid chromatography purity of at least 99%. The desired concentrations of citral solution were freshly prepared in 0.1% dimethyl sulfoxide before use. All other chemicals were of analytical grade and were unaltered.

### Bacterial Strains and Growth Conditions

*V. parahaemolyticus* strains ATCC 17802 and ATCC 33847 (American Type Culture Collection, Manassas, USA) were used in this study. A further 46 *V. parahaemolyticus* isolates recovered from marine products collected by the Hong Kong Polytechnic University were also included. A loopful of each strain was inoculated into 30 ml of tryptic soy broth (TSB; Difco, Franklin Lakes, NJ, USA) containing 3% (wt/vol) NaCl and incubated at 37°C. Subsequently, the *V. parahaemolyticus* cultures were centrifuged at 8,000 × *g* for 5 min at 4°C, washed, and then re-suspended in fresh TSB (3% NaCl). *Vibrio harveyi* strains BB170 and BB120 (ATCC) were used for the detection of AI-2 in QS inhibition assays.

### Minimum Inhibitory Concentration Assay

The minimum inhibitory concentrations (MICs) of citral against two *V. parahaemolyticus* ATCC strains (17802 and 33847) and four isolates were determined using the agar dilution method (Li et al., 2014). Different concentrations of citral (from 25 to 400 μg/ml) were mixed with warm tryptone soya agar (TSA) supplemented with 3% NaCl and transferred into sterile 24-well plates. Aliquots (2 μl) of *V. parahaemolyticus* suspension (~10^5^ colony-forming units, CFU) were then spotted onto the medium, and the samples were incubated at 37°C for 24 h. The MIC was defined as the minimum concentration of citral at which there was no visible growth of the test strain. Kanamycin (100 mg/L) was used as a positive control. Results were calculated as a mean of experiments performed in triplicate.

### Sub-inhibitory Concentration Assay

A broth dilution assay (Shi et al., 2017) with slight modifications was performed to identify the concentrations of citral that had no effect on the growth of *V. parahaemolyticus*. Equivalent volumes (125 μl) of bacterial suspension (~10^4^ CFU) and citral solution were transferred into 96-well microtiter plates to give final citral concentrations of 0 (control), 3.125, 6.25, 12.5, 25, 50, 100, and 200 μg/ml. The samples were incubated for 24 h at 37°C, and cell density was measured as optical density (OD) at 600 nm using an automatic growth curve analyzer (Labsystems, Helsinki, Finland). Medium without bacteria was used as the negative control. OD measurements were taken in triplicate from three independent experiments to obtain a mean value for each citral concentration.

### Sub-inhibitory Concentration and Autoinducer-2 Determination Assay

To detect and quantify AI-2 production by *V. parahaemolyticus*, a bioluminescent bacterial reporter strain called *V. harveyi* BB170, which produces light in response to AI-2, was used in the assay (Han et al., 2016). The sub-inhibitory concentration (SIC) of citral for *V. harveyi* BB170 was first determined as described previously. The effect of citral on AI-2 production was then determined according to a previously described method (Han et al., 2016), with minor modifications. Briefly, a log-phase culture of *V. parahaemolyticus* ATCC 17802 was centrifuged (8,000 × *g*, 5 min, 4°C) and the cell pellet was re-suspended in TSB to an OD_600 nm_ = 0.5. The suspension was then treated with 3.125 or 6.25 μg/ml of citral and incubated with shaking for 6 h at 30°C. Supernatant containing QS molecules was obtained by centrifuging the cultures at 8,000 × *g* for 10 min at 4°C. The supernatants were passed through 0.2-μm Tuffryn syringe filters and stored at −20°C. The cell-free supernatants were then tested for the presence of autoinducers that could induce luminescence in *V. harveyi* reporter strain BB170. For this bioassay, *V. harveyi* strain BB170 was grown overnight at 30°C with aeration in autoinducer bioassay (AB) broth and then diluted 1:1,000 in AB medium. Diluted strain BB170 was then added along with each individual cell-free supernatant to 50-ml tubes and incubated for 16 h at 30°C with shaking at 180 × *g* to allow the development of luminescence by the reporter strain. Aliquots (100 μl) of each of the samples were transferred to 96-well white microtiter plates and luminescence was measured using a microplate reader (Tecan, Infinite M200 PRO, Männedorf, Switzerland). *V. harveyi* strain BB120 (which produces AI-1 and AI-2) was used as a positive control and was grown overnight at 30°C with shaking in LB broth, following which, 1 ml of cell-free supernatant from the culture was prepared as described previously.

### Motility Assay

Swimming and swarming motility were assessed as described by Packiavathy et al. (2012). For the swimming motility assay, 20 ml of LB broth containing 0.3% (wt/vol) agar was used. Citral was added to the warm (45°C) semi-solid agar medium to obtain final concentrations of 0, 3.125, 6.25, and 12.5 μg/ml. After being dried for 1 h, the semi-solid agar plates were spotted with 5-μl volumes of *V. parahaemolyticus* culture (~1 × 10^6^ CFU, at the center of the plate) and incubated at 37°C for 7 h. To examine swarming motility, 20 ml of LB broth was supplemented with 0.5% (wt/vol) agar and 0.5% (wt/vol) glucose and mixed with citral at final concentrations of 0, 3.125, 6.25, and 12.5 μg/ml. Aliquots (5 μl) of bacterial culture were then stabbed into the medium and plates were incubated upside down at 37°C for 20 h. The diameter of the motility area was measured using AutoCAD. Medium without citral was used as a control.

### Congo Red Agar Assay

To visually detect biofilm formation by the 46 *V. parahaemolyticus* isolates, a Congo red agar (CRA) assay was performed as previously described by Wojtyczka et al. (2014). The isolates were inoculated onto brain-heart infusion (BHI) agar medium supplemented with 5% (wt/vol) sucrose and Congo red, samples were incubated for 24 h at 37°C. Under these conditions, biofilm producers form black crusty colonies on the CRA-BHI plates, whereas non-producers form red colonies.

### Crystal Violet Assay

Biofilm formation was also examined with respect to biomass using a Crystal violet (CV) staining method described by Naves et al. (2008), with some modifications. Log-phase cultures of *V. parahaemolyticus* ATCC 17802, VP244, and VP253 were separately centrifuged (8,000 × *g*, 5 min, 4°C) and re-suspended in TSB (3% NaCl). Then, 250-μl aliquots of the cell suspensions (OD_600 nm_ = 1) supplemented with citral (0, 3.125, 6.25, and 12.5 μg/ml) were inoculated into sterile 96-well polystyrene tissue culture plates and incubated for 24, 48, or 72 h without agitation. Uninoculated LB broth was used as a control. Six wells were used per strain. At each time point, bacterial growth was determined by measuring the OD at 630 nm using a microplate spectrophotometer (model 680; Bio-Rad, Hercules, CA, USA). Growth medium was then carefully removed, and each well was rinsed once with distilled water to remove any unbound bacteria. After being air-dried for 30 min, biofilms were stained with 250 μl of 1% (wt/vol) CV solution (Tianjin Kermel Chemical Regent Co., Tianjin, China) for 20 min at room temperature. The CV dye was then discarded, and the wells were rinsed three times with distilled water to remove any unbound colorant. After drying, the stained biofilm was solubilized in 250 μl of 33% (vol/vol) glacial acetic acid for 20 min and the OD_570 nm_ was measured. The specific biofilm formation (SBF) was calculated by attaching and stained bacteria (OD_570 nm_) normalized with cell growth (OD_630 nm_). The experiment was repeated at least three times.

### Field Emission Scanning Electron Microscopy

Field emission scanning electron microscopy (FE-SEM) was used to assess the effect of citral on the biofilm morphology of *V. parahaemolyticus* ATCC 17802 as described previously (Li et al., 2014), with some modifications. Bacterial cells (OD_600 nm_ = 1) were treated with citral (0, 3.125, 6.25, or 12.5 μg/ml) and then incubated at 30°C for 72 h to allow biofilm formation on coverslips. Bacterial suspensions were removed and then the coverslips were gently washed twice with phosphate-buffered saline (PBS, pH = 7.0) before the addition of 2.5% (vol/vol) glutaraldehyde and incubation overnight at 4°C to fix the cells. The samples were then serially dehydrated with ethanol (30, 50, 70, 80, 90, and 100%) for 10 min each. The samples were sputter-coated with gold under vacuum conditions and visualized using a scanning electron microscope (S-4800; Hitachi, Tokyo, Japan).

### Adhesion of Caco-2 Cells

Human colon adenocarcinoma cell line Caco-2 was maintained in Dulbecco’s Modified Eagle Medium (DMEM) (Gibco, Grand Island, NY, USA) supplemented with 10% (vol/vol) fetal bovine serum (Hyclone, Logan, UT, USA), 1% (vol/vol) non-essential amino acids (Gibco), and 1% (vol/vol) double antibiotic solution (100 U/ml penicillin and 100 μg/ml streptomycin, Hyclone). Maintenance of the cell lines and subsequent experiments were carried out at 37°C in a humidified atmosphere containing 5% CO_2_.

To evaluate the effect of citral on the adhesion of *V. parahaemolyticus* ATCC 17802 to Caco-2 cells, an adhesion assay was performed as described previously (Amalaradjou et al., 2014). Caco-2 cells were seeded in 24-well tissue culture plates (10^5^ cells/well) containing supplemented DMEM and incubated for 24 h. *V. parahaemolyticus* ATCC 17802 was grown to log phase with and without SICs of citral (3.125, 6.25, or 12.5 μg/ml), centrifuged, and then re-suspended in cell culture medium without antibiotics. The Caco-2 cells were rinsed then with PBS before the addition of culture medium containing the *V. parahaemolyticus* suspension (10^7^ CFU, MOI = 10). The tissue culture plates were centrifuged at 600 × *g* for 5 min and incubated at 37°C in a humidified 5% CO_2_ incubator for 2 h. The culture medium was removed, then the infected monolayer cells were rinsed three times with PBS, and lysed with 0.1% Triton X-100 (Amresco, Solon, OH, USA). The number of viable adherent *V. parahaemolyticus* cells was determined by serial dilution and plating on TSA (3% NaCl) plates.

### Quantitative Real-Time Polymerase Chain Reaction

The effects of citral on the transcription of *V. parahaemolyticus* virulence genes (*flaA*, *flgM*, *flgL*, *ompW*, *VP0950*, *VP0952*, *VP0962*, *luxS*, *aphA*, *vopQ*, *vpA0450*, and *toxR*) and antimicrobial peptide (AMP)-resistant genes (*tolC*, *nusA*, *atpA*, *dld* and *fla*),were examined using a real-time quantitative polymerase chain reaction (RT-qPCR) assay. Total RNA was extracted from log-phase bacterial cultures grown with and without SICs of citral using a RNAprep Pure Bacteria Kit (Tiangen, Beijing, China) according to the manufacturer’s protocol. After measuring RNA concentrations using a nucleic acid and protein spectrophotometer (Nano-200; Aosheng Instrument Co., Hangzhou, China), cDNA was synthesized using a PrimeScript RT Reagent Kit (Takara, Kyoto, Japan) according to the manufacturer’s instructions. Primers used for RT-qPCR are listed in Table 1. RT-qPCR reactions were carried out in a 25-μl reaction volume using SYBR Premix Ex Taq II (Takara). The thermal cycler parameters were 95°C for 30 s, 40 cycles of 95°C for 5 s, and 60°C for 30 s, followed by dissociation steps of 95°C for 15 s and 60°C for 30 s. All samples were analyzed in triplicate and normalized to the endogenous control (*puvA*) gene (Coutard et al., 2007). Samples were run on an IQ5 system (Bio-Rad), and the transcription of target genes versus *puvA* was determined as previously described (Shi et al., 2017).

**Table 1:** Effects of sub-inhibitory concentrations of citral on the transcription of virulence-associated genes and antibiotic resistance genes in *Vibrio parahaemolyticus* ATCC 17802.

Target gene	Sequence of primers (5′-3′)^c^	Relative gene expression	
		12.5 μg/ml	6.25 μg/ml
*puvA*	F, CAAACTCACTCAGACTC R, CGAACCGATTCAACAC	1	1
*tolc*	F, CGCAACTCGTCGCCTAT R, TGTCTTGTTCGCTTAGTGTACCA	−3.05 ± 0.41^**^	−1.12 ± 0.32^**^
*fla*	F, AGATCGGTTTTGGTGATGC R, CGTTGGCTGTCTACTGATTTAAG	−1.85 ± 0.19^**^	−1.17 ± 0.03^**^
*atpA*	F, CGATGATCTATCTAAACAAGCGG R, TAAGTAGAATACGTCACCTGGGA	−1.46 ± 0.43^**^	−1.26 ± 0.04^**^
*nusA*	F, TGTTTATCACTCGTTCTAAGCCT R, GTTTGTCATTTGTTTTCACTGCG	−2.18 ± 0.28^**^	−1.45 ± 0.12^**^
*flaA*	F, CGGACTAAACCGTATCGCTGAAA R, GGCTGCCCATAGAAAGCATTACA	−9.27 ± 1.05^**^	−2.24 ± 0.14^*^
*flgL*	F, CGTCAGCGTCCACCACTT R, GCGGCTCTGACTTACTGCTA	−1.35 ± 0.48^**^	−1.27 ± 0.37^**^
*flgM*	F, CGTCAGCGTCCACCACTT R, GCGGCTCTGACTTACTGCTA	−1.57 ± 0.48^**^	−1.52 ± 0.03^**^
*dld*	F, TTATCCCGCATGAAGACCC R, CCGATGATACCACCGCC	−2.06 ± 0.07^**^	−1.35 ± 0.04^**^
*luxS*	F, GGATTTTGTTCTGGCTTTCCACTT R, GGGATGTCGCACTGGTTTTTAC	−9.17 ± 2.01^**^	−6.46 ± 1.41^**^
*ompW*	F, TCGTGTCACCAAGTGTTTTCG R, CGTGGCTGAATGGTGTTGC	−5.51 ± 0.49^**^	−1.38 ± 0.12^**^
*vopQ*	F, CCACAAGTTTGCTTCGGTTAG R, GGTTCTCCTCGGTAGCCTGA	−2.53 ± 0.22^**^	−2.34 ± 0.05^**^
*vp0950*	F, GCCAAACTTCTCAAACAACA R, ATGAAACGCAATTTACCATC	−10.64 ± 0.63^**^	−3.50 ± 0.31^**^
*vp0952*	F, TATGATGGTGTTTGGTGC R, TGTTTTTCTGAGCGTTTC	−2.43 ± 0.18^**^	−1.49 ± 0.06^**^
*vp0962*	F, GACCAAGACCCAGTGAGA R, GGTAAAGCCAGCAAAGTT	−5.74 ± 0.84^**^	−1.46 ± 0.22
*vpA0450*	F, TTGCTGAAGGCTCTGATG R, CTGCACTGGCTTATGGTC	−2.79 ± 0.51^**^	−1.93 ± 0.19^**^
*aphA*	F, ACACCCAACCGTTCGTGATG R, GTTGAAGGCGTTGCGTAGTAAG	−1.21 ± 0.38^**^	−1.17 ± 0.02^**^
*toxR*	F, ACAATGACGCCTCTGCTAAT R, ACTCACCAATCTGACGGAACT	−2.87 ± 0.87^**^	−1.82 ± 0.15^**^

* *p* ≤ 0.05;

** *p* ≤ 0.01;

c F, forward; R, reverse.

### Statistical Analysis

All experiments were performed at least in triplicate. Statistical analyses were performed using SPSS software (version 19.0; SPSS, Inc., Chicago, IL, USA). The data were presented as the mean values ± SD (*n* = 3) and differences between means were tested by Student’s *t*-test. Differences were considered significant at *p* ≤ 0.05.

## Results

### Determination of Minimum Inhibitory Concentrations

The MICs of citral for six *V. parahaemolyticus* strains ranged from 100 to 300 μg/ml (Table 2). *V. parahaemolyticus* ATCC 17802 was the most susceptible to citral (MIC = 100 μg/ml), while the four *V. parahaemolyticus* isolates showed a three-fold higher tolerance to citral (MIC = 300 μg/ml).

**Table 2:** Minimum inhibitory concentrations (MICs) of citral against different strains of *Vibrio parahaemolyticus*.

Strain	Origin	MICs (μg/ml)
ATCC 17802	Shirasu food poisoning	100
ATCC 33847	Gastroenteritis	150
VP240	Marine products	300
VP245	Marine products	300
VP247	Marine products	300
VP248	Marine products	300

### Determination of Sub-inhibitory Concentration and Inhibition of Autoinducer-2 Quorum Sensing Signaling

**Figure 1:**
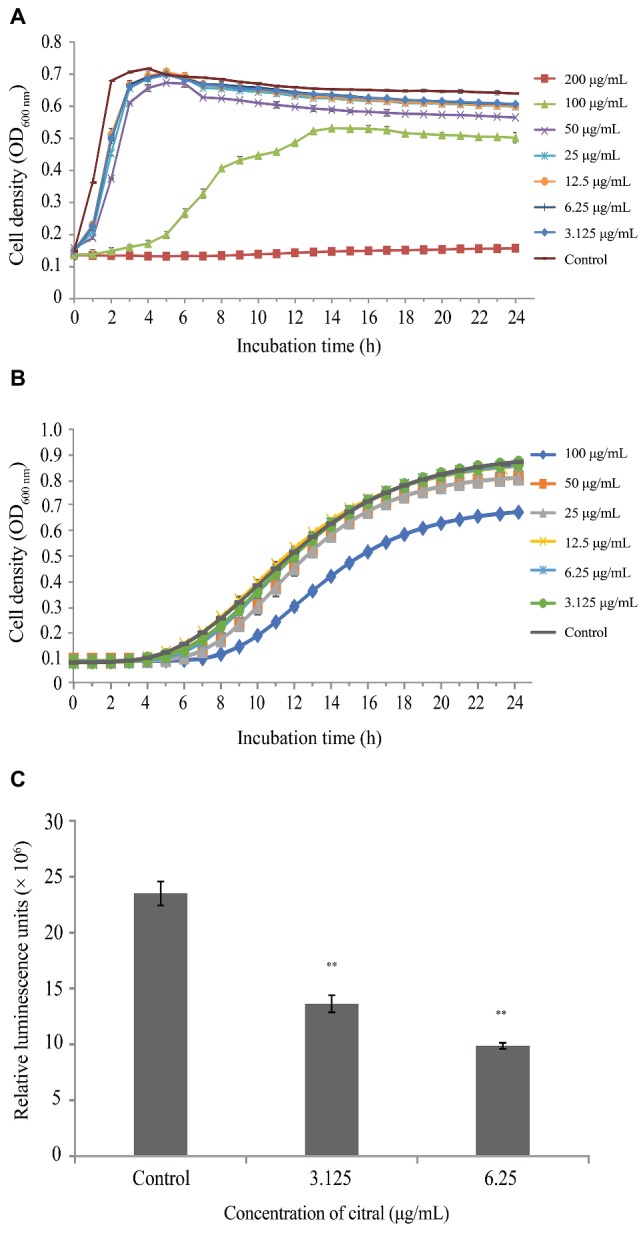
**(A)** Growth of *Vibrio parahaemolyticus* ATCC 17802 treated with different concentrations of citral. Each value represents the average of three independent measurements. **(B)** Growth of *Vibrio harveyi* BB170 treated with different concentrations of citral. Each value represents the average of three independent measurements. **(C)** Inhibition of AI-2 production by *Vibrio harveyi* BB170 at sub-inhibitory concentrations of citral. Bars represent the standard deviation (*n* = 3). ^**^ *p* ≤ 0.01.

The growth of *V. parahaemolyticus* was suppressed at concentrations of citral above 12.5 μg/ml (Figure 1A). Therefore, 3.125, 6.25, and 12.5 μg/ml were chosen as the SICs for further virulence-related assays.

At concentrations below 6.25 μg/ml, citral exhibited no inhibition of *V. harveyi* strain BB170 growth (Figure 1B). Production of AI-2 by *V. parahaemolyticus* ATCC 17802 was reduced by 42 and 58% following exposure to 3.125 and 6.25 μg/ml of citral, respectively (*p* ≤ 0.01) (Figure 1C).

### Inhibition of Swimming and Swarming Motility

**Figure 2:**
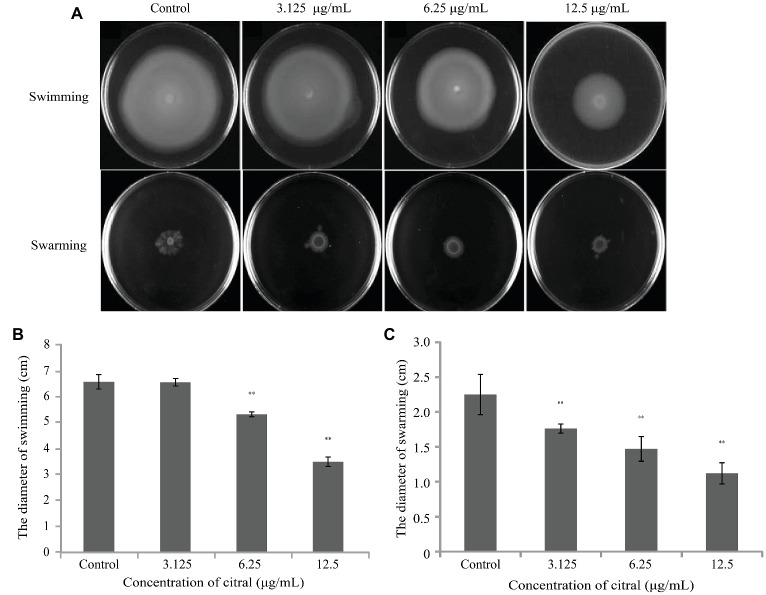
**(A)** The effects of sub-inhibitory concentrations of citral on the swimming and swarming motility of *Vibrio parahaemolyticus* ATCC 17802. **(B)** Measurement of *V. parahaemolyticus* ATCC 17802 migration in swimming motility assay. **(C)** Measurement of *V. parahaemolyticus* ATCC 17802 migration in swarming motility assay. The diameter is presented as the mean ± the standard deviation of three independent experiments. ^**^ *p* ≤ 0.01.

Citral effectively reduced the swimming and swarming motility (Figure 2) of *V. parahaemolyticus* in a concentration-dependent manner. At 6.25 and 12.5 μg/ml, citral significantly decreased the diameter of the swimming area by 20 and 47%, respectively (*p* ≤ 0.01), while 3.125, 6.25, and 12.5 μg/ml of citral significantly decreased the swarming area by 22, 35, and 50%, respectively (*p* ≤ 0.01).

### Reduction in Biomass

Only two of the 46 tested marine product-derived *V. parahaemolyticus* isolates (VP244 and VP253) formed black crusty colonies on CRA medium, indicating biofilm formation.

**Figure 3:**
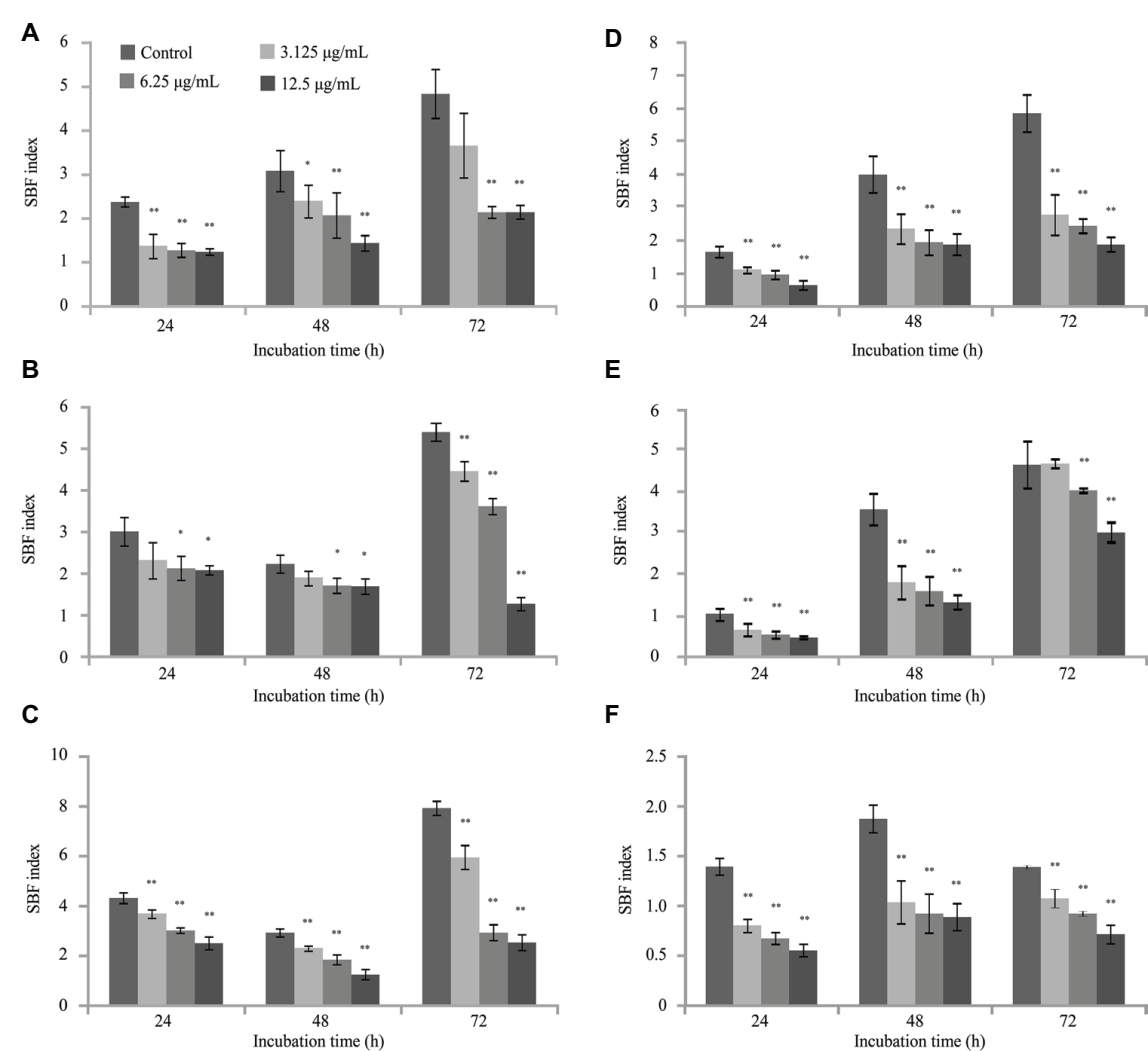
The effects of citral on biofilm formation by *Vibrio parahaemolyticus* ATCC 17802 **(A,D)**, VP244 **(B**,**E)** and VP253 **(C,F)** at 20°C **(A–C)** and 30°C **(D–F)**. Bars represent the standard deviation (*n* = 3). *p ≤ 0.05, ^**^ *p* ≤ 0.01.

The SBF of VP253 at 30°C was lower than that at 20°C, while the other two strains (ATCC 17802 and VP244) were not affected by the temperature change (Figure 3). At concentrations of 3.125, 6.25, and 12.5 μg/ml, citral significantly (*p* ≤ 0.05) reduced biofilm formation by *V. parahaemolyticus* strains ATCC 17802, VP244, and VP253 at both 20 and 30°C in both a concentration- and time-dependent manner. Following incubation for 72 h, citral (12.5 μg/ml) caused a greater decrease in *V. parahaemolyticus* ATCC 17802 biofilm density at 30°C (67.97% reduction compared with no-citral control) (Figure 3D) than at 20°C (55.73% reduction) (Figure 3A). In contrast, a greater decrease was observed for strains VP244 and VP253 at 20°C (Figures 3B,C) compared with at 30°C (Figures 3E,F).

### Observed Changes in Biofilm Structure Following Citral Treatment by Field Emission Scanning Electron Microscopy

**Figure 4:**
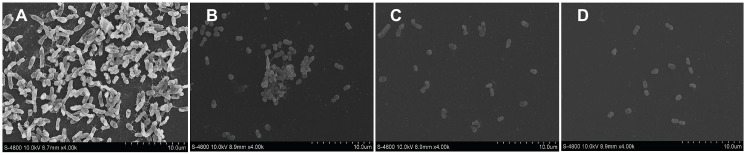
The effects of SICs of citral *Vibrio parahaemolyticus* ATCC 17802 as observed by field-emission scanning electron microscopy (4,000 × magnification). **(A–D)** Control, 3.125 μg/ml, 6.25 μg/ml, and 12.5 μg/ml citral, respectively.

The architectural integrity of biofilm and the aggregation of cells were significantly altered following exposure to citral (3.125, 6.25, or 12.5 μg/ml) (Figure 4). Biofilm formed by *V. parahaemolyticus* control cultures displayed firm three-dimensional, multicellular, complex, self-assembled structures that contained extracellular polymeric substances (EPS) (Figure 4A). With increasing concentrations of citral, the *V. parahaemolyticus* cells secreted a lesser amount of EPS and the biofilm structure became loose (Figures 4B,C). At a citral concentration of 12.5 μg/ml, the biofilm structure was completely disrupted, with individual, dispersed cells (Figure 4D).

### Interruption of *V. parahaemolyticus* Adhesion to Caco-2 Cells

**Figure 5:**
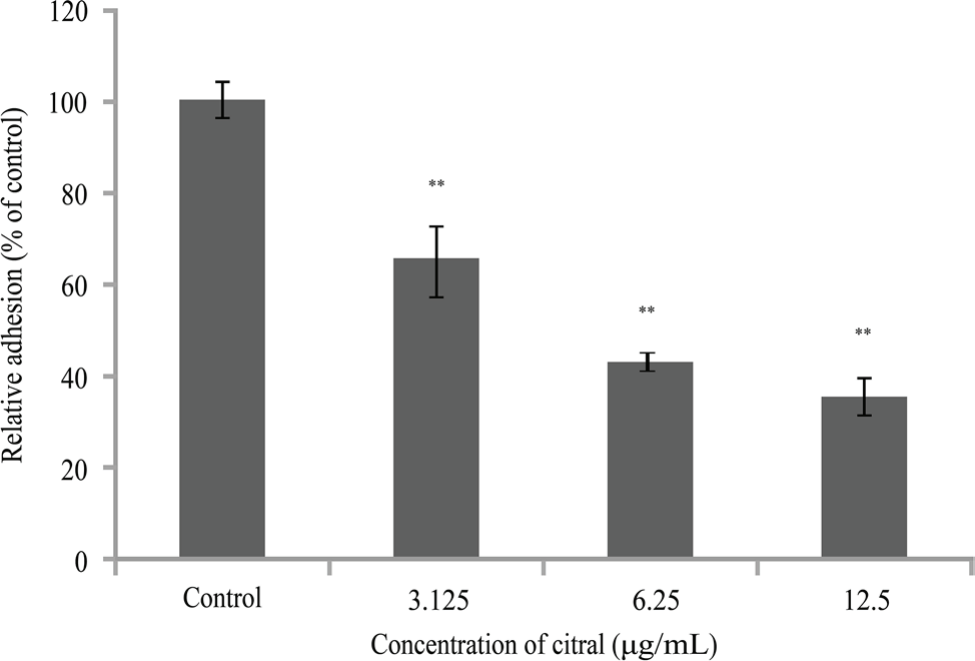
The effects of sub-inhibitory concentrations of citral on adhesion of *Vibrio parahaemolyticus* ATCC 17802 to Caco-2 cells. Bars represent the standard deviation (*n* = 3). ^**^ *p* ≤ 0.01.

Citral significantly (*p* ≤ 0.01) inhibited the ability of *V. parahaemolyticus* to adhere to Caco-2 cells in a dose-dependent manner (Figure 5). The adherence of *V. parahaemolyticus* cells pre-exposed to 3.125, 6.25, or 12.5 μg/ml of citral was reduced by 35, 59, and 65%, respectively, compared with the control (*p* ≤ 0.01).

### Down-regulation of Virulence-Associated Genes and Antimicrobial Peptide-Resistant Genes

Citral downregulated the transcription of genes associated with flagella regulation (*flaA*, *flgM*, *flgL*), biofilm formation (*ompW*, *vp0950*, *vp0952*, *vp0962*), QS regulation (*luxS*, *aphA*), T3SS1 (*vopQ*, *vpA0450*), toxin production (*toxR*), and AMP-resistant genes (*tolC*, *nusA*, *atpA*, *dld* and *fla*) to various degrees (Table 1). Among these, the most significant effects were observed following treatment with 12.5 μg/ml of citral, with greater than nine-fold decreases in *flaA*, *luxS*, and *vp0950* transcription.

## Discussion

The global disease outbreaks and food contamination caused by *V. parahaemolyticus* underscore the importance of controlling the expression of virulence factors by this important foodborne pathogen. Citral has previously been shown to effectively control separate and specific virulence factors in different pathogens (Echeverrigaray et al., 2008; Ahmad et al., 2015). However, the effect of citral on virulence factors and antimicrobial resistance of *V. parahaemolyticus* still needs to be adequately investigated.

In previous studies, the MIC of citral was found to be 584 μg/ml for *Cronobacter sakazakii* (Shi et al., 2017), while curcumin inhibits *V. parahaemolyticus* and other *Vibrio* spp. with MICs exceeding 150 μg/ml (Packiavathy et al., 2013). In the current study, citral showed effective antibacterial activity against *V. parahaemolyticus* strains, with MICs ranging from 100 to 300 μg/ml.

*V. parahaemolyticus* can alternate between two cell types depending on the growth conditions (Zhu et al., 2013). When grown in low viscosity and liquid culture, the cells appear as short rods with a single-sheathed polar flagellum, which is used for swimming and overcoming repulsive forces between the bacteria and the host tissues. However, when grown on solid surfaces and in high viscosity medium, the cells switch to a swarmer cell type and utilize their lateral flagellum to increase the surface tension, allowing them to aggregate and form a biofilm. FlaA, a specific polar flagellin, can mediate the formation of the flagellar filament, resulting in swimming motility (Loh et al., 2004). FlgL is the hook-associated protein 2 that plays an important role in polar flagellation and adhesion to host cells (Kim et al., 2008). FlgM is an anti-σ factor, which can mirror the favorable conditions for swarming motility, resulting in an increase in flagellar filament numbers and switching the cell type to swarmer (Zhu et al., 2013). Echeverrigaray et al. (2008) demonstrated that citral effectively inhibited the swarming ability of *Proteus mirabilis*. In this study, citral effectively repressed both swarming and swimming motility of *V. parahaemolyticus*. Moreover, citral downregulated the transcription of *flaA*, *flgL*, and *flgM*. It was hypothesized that citral reduced the secretion of FlaA, FlgM, and FlgL, which impeded the ability of *V. parahaemolyticus* to recognize favorable attachment surfaces and the biosynthesis of polar and lateral flagella. Biofilm formation may be a major factor in the lowered shelf-life of *V. parahaemolyticus*-contaminated seafood and aid in the transmission of disease (Mizan et al., 2016). The CV assay showed that citral reduced the biofilm biomass of *V. parahaemolyticus* in a concentration-dependent manner at both 20 and 30°C within 3 days. A previous study showed that temperature influenced the production of EPS, which is related to biofilm formation (Garrett et al., 2008). In this study, the biofilm formation of isolate VP253 was decreased at 30°C, possibly because the isolate was more sensitive to the high temperature. Moreover, citral caused greater biofilm biomass reduction of *V. parahaemolyticus* isolates VP244 and VP253 at 20°C, while it showed more effective inhibition of biofilm formation by *V. parahaemolyticus* ATCC 17802 at 30°C. It could be due to the fact that the optimal temperature for EPS secretion of *V. parahaemolyticus* ATCC 17802 might be 30°C, while for the two other isolates, 20°C may be the optimal temperature. In line with the CV results, the FE-SEM images showed that the structure of the *V. parahaemolyticus* biofilm was obviously affected by the treatment of citral. The VP0950 (encoding a lipoprotein-related protein), VP0952, and VP0962 (encoding hypothetical proteins) were parts of biofilm (Boyd et al., 2008). The outer membrane proteins (OMPs) play an important role in nutrient uptake and in interactions with the environment and host tissues (Ritter et al., 2012). The transcription of *ompW* was related to the biofilm formation in *Pseudoalteromonas* sp. D41 (Ritter et al., 2012). In this study, citral impeded biofilm development of *V. parahaemolyticus* strains, possibly by damaging the biosynthesis of biofilm-associated proteins, repressing the expression of OMP-associated genes (such as *ompW*) and therefore the transportation of substances associated with the biofilm formation. Bacterial adherence to epithelial cell surfaces is a key stage in their survival and colonization of the gastrointestinal tract (Rasko and Sperandio, 2010). We found that the number of *V. parahaemolyticus* cells adhered to Caco-2 cells was decreased after pretreatment with citral. This finding is in agreement with the results of Shi et al. (2017), who showed that citral effectively inhibited the adhesion of *C. sakazakii* ATCC 29544 to Caco-2 cells. Additionally, Kirov (2003) reported that lateral flagella of *Aeromonas* strains caused persistent and dysenteric infections in the gastrointestinal tract. It was likely that the inhibition of flagella biosynthesis by citral contributed to the attenuation of *V. parahaemolyticus* adherence to Caco-2 cells.

T3SS1 effectors help *V. parahaemolyticus* to evade the host immune response, inducing autophagy followed by efficient lysis of the infected host cells, as well as causing cytotoxicity in host cells (Park et al., 2004). Among these effectors, VopQ causes rapid induction of autophagy in target cells, while VPA0450 destabilizes the cell by interfering with the association between the actin cytoskeleton and the cell membrane (Zhang and Orth, 2013). In this study, citral effectively downregulated the transcription of genes coding for the VopQ and VPA0450 effectors. As a result of citral-induced inhibition of these important effectors, *V. parahaemolyticus* might be more easily eliminated by the host immune response and find it more difficult to invade host cells. Additionally, flagella contain a sophisticated export apparatus involved in the secretion of several virulence factors that is closely related to type III secretion pathways (Kirov, 2003). The damage to the flagella structure caused by citral may influence type III secretion pathways, thereby impeding the delivery of effectors.

ToxR coordinately regulates several virulence-associated genes, including the *tcp* genes (toxin-coregulated pilus) and the *ompU* and *ompT* genes (major outer membrane proteins) in *Vibrio cholerae* (Lee et al., 2000; Childers and Klose, 2007). Moreover, Vp-ToxR directly promoted the expression of *tdh2* and resulted in the development of Kanagawa phenomenon-positive virulent strains (Lin et al., 1993). Thermostable direct hemolysin (TDH) is a protein toxin that has several biological functions, including hemolytic, enterotoxic, and cytotoxic activities (Lin et al., 1993). We observed that citral effectively downregulated the expression of *toxR* in *V. parahaemolyticus*, which possibly reduced the secretion of TDH or other toxins.

AI-2, a dihydroxy pentanedione-derived molecule synthesized by LuxS-like synthases, plays a role in inter-species communication in a wide variety of bacteria. At the low cell density, AphA is increasingly expressed to trigger the transcription of virulence genes which associated with infection (Wang et al., 2013). In this study, citral effectively repressed the biosynthesis of AI-2 and the transcription of *luxS* and *aphA* in *V. parahaemolyticus*. Similarly, a minimum citral concentration of 0.016 mg/ml inhibited QS by *Pseudomonas aeruginosa* (Ahmad et al., 2015). Low concentrations (100 μmol/L) of cinnamaldehyde were also effective at inhibiting AI-2-mediated QS in *V. harveyi* BB170 (Niu et al., 2006).

A *luxS* null mutation was reported to eliminate growth-phase-dependent control of *flaA* in *Helicobacter pylori* and to downregulate *flgM* transcription in *Escherichia coli* K12 (Loh et al., 2004; Ling et al., 2010), while AphA is associated with the biofilm formation and motility in *V. parahaemolyticus* (Wang et al., 2013). Moreover, QS appeared to repress ToxR-regulated virulence genes in *V. cholerae* (Zhu et al., 2002). In the present study, determining the effects of citral on multiple virulence targets could not exclude the influence of QS. The reduction of AI-2 may contribute to the changes in some traits during citral treatment, but whether it is the determining factor needs further investigation.

The growing emergence of antimicrobial-resistant *V. parahaemolyticus* becomes a challenge of controlling *V. parahaemolyticus* infections and food contamination (Xie et al., 2016). The OMPs (TolC and flagellin), transcription termination factor (NusA), ATP synthase F1, alpha subunit (F1-ATPa), and dihydrolipoamide dehydrogenase (DLD) were associated with AMP-resistance (Shen et al., 2010). This study showed that citral downregulated multiple AMP-resistant genes (*tolC*, *nusA*, *atpA*, *dld* and *fla*) to various levels. It could be speculated that citral played a role in damaging the multidrug efflux transporter and membranes of *V. parahaemolyticus*, therefore reduced the resistance of *V. parahaemolyticus*.

## Conclusions

In conclusion, this investigation indicated that citral attenuated multiple virulence factors of *V. parahaemolyticus*, including QS, motility, biofilm formation, the adhesion to Caco-2 cells, and repressed the expression of genes related to flagella (polar and lateral), biofilm and T3SS1 effectors, virulence regulators (*luxS*, *aphA* and *toxR*), and AMP resistance. The results obtained in this work demonstrate the inhibitory effect of citral on virulence factors of *V. parahaemolyticus*. However, the data reported in this study only demonstrate the anti-virulence effect of citral *in vitro*. Further research is needed to clarify the mode of the anti-virulence action of citral and to investigate its effects in experimental animal models, aiming toward the application of citral as an alternative strategy to control the infections of *V. parahaemolyticus*.

## Author Contributions

CS and YS conceived and designed the experiments. DG, ZH and HS performed the experiments. ZZ analyzed the data. XX contributed reagents, materials, and analysis tools. YS and CS wrote the manuscript.

### Conflict of Interest Statement

The authors declare that the research was conducted in the absence of any commercial or financial relationships that could be construed as a potential conflict of interest.
